# The double-edged sword of rotational shift work: a qualitative study of worker perceptions in Saudi Arabia

**DOI:** 10.3389/fpsyg.2026.1871679

**Published:** 2026-09-18

**Authors:** Mohammad A. Algarni, Omar A. Maash

**Affiliations:** 1 Department of Human Resources, King Abdulaziz University, Jeddah, Saudi Arabia; 2 Department of Management Science, Royal Commission for Jubail and Yanbu, Yanbu Industrial College, Yanbu, Saudi Arabia

**Keywords:** circadian disruption, job demands-resources model, occupational psychology, petrochemical industry, psychosocial well-being, qualitative phenomenology, rotational shift work, Saudi Arabia

## Abstract

**Background:**

Rotational shift work is a defining feature of 24/7 industries, yet its psychological consequences remain poorly understood, particularly outside Western healthcare contexts.

**Methods:**

Grounded in the Job Demands-Resources (JD-R) framework, this qualitative phenomenological study examined how rotational shift schedules shape the psychological well-being, work-life balance, and occupational outcomes of 16 shift workers in Saudi Arabia’s petrochemical sector, selected through purposive sampling and interviewed using semi-structured in-depth interviews; data were analyzed using Braun and Clarke’s (2019) six-phase reflexive thematic analysis.

**Results:**

Three overarching themes emerged: significant health effects including chronic sleep disruption, circadian rhythm misalignment, physical exhaustion, and erosion of healthy behavioral routines; complex work-life balance negotiations shaped by financial necessity, personal flexibility, social isolation, and uniquely in this cultural context, disruption to religious obligations including prayer schedules and Ramadan practices; and mixed occupational outcomes whereby rotational shifts enhanced decision-making autonomy and experiential learning while simultaneously constraining career progression, undermining professional visibility, and creating tension in workplace relationships.

**Conclusion:**

These findings extend JD-R theory into a non-Western, non-healthcare shift work context, revealing that culturally specific demands, particularly religious and social obligations, constitute a distinct and underexamined category of psychosocial burden, with implications for organizational policies addressing sleep hygiene, religious accommodation, and structured career development for shift workers.

## 1 Introduction

The psychological experience of work is fundamentally shaped by how time is structured, and for a growing proportion of the global workforce, that structure is one of perpetual rotation. Time management and optimal utilization of time are fundamental to the efficient achievement of personal, business, societal, and governmental objectives. In response to growing global demands, most sectors worldwide have extended operational hours through continuous rotational shift systems. This transformation was initially driven by the healthcare sector, particularly nursing, in which 24-h patient care is necessary. Subsequently, significant business developments worldwide have compelled companies and employers to adopt variable shift systems, enabling continuous operations 24 h a day, 7 days a week. However, shift schedules may prove unsuitable for employees, depending on their personal circumstances.

Despite these challenges, the literature reveals that rotational work can offer numerous advantages from both business and worker perspectives. For instance, some shift workers prefer shift schedules for practical reasons such as childcare arrangements, educational pursuits, enhanced compensation, or personal preference. However, the fact remains that many employees work shifts because it represents their only available employment option (McMenamin, 2007). Certain occupations are inherently associated with shift work due to their operational nature, particularly those providing 24/7 services such as healthcare professionals, police officers, and emergency responders. Similarly, manufacturing industries often operate continuously, requiring round-the-clock staffing (Williams, 2008).

Despite the practical advantages of shift work, there is a substantial body of research that highlights the negative effects of rotational work on employees’ well-being. Critics argue that shift work adversely impacts health, sleep patterns, and social relationships (Emekli, 2022). Additional concerns include uncontrollable weight fluctuations and disrupted appetite regulation (Vidafar et al., 2020). Caruso (2014) identified five critical poor health behaviors linked to rotational work: shortened sleep duration, increased smoking and obesity, reduced physical activity, and elevated alcohol consumption. From a psychological standpoint, these outcomes are well captured by the Job Demands-Resources (JD-R) model (Demerouti et al., 2001), which proposes that job demands such as irregular schedules, sleep deprivation, and social disruption deplete workers’ psychological and physical resources, increasing the risk of burnout and reduced well-being. Conversely, job resources such as financial incentives, scheduling autonomy, and collegial support may buffer these negative effects, motivating engagement even under demanding conditions. This framework provides a useful theoretical lens through which to examine shift workers’ lived experiences.

Given these conflicting perspectives, it becomes crucial for organizations to understand their employees’ viewpoints regarding shift work preferences. Organizations must explore whether employees favor shift work systems or fixed-hour schedules, while identifying the underlying motivators influencing these preferences. Financial incentives often drive shift work preference, whereas concerns about physical, psychological, or social impacts, particularly regarding night shifts, may favor fixed schedules (Emekli, 2022). This understanding is essential for organizations seeking to minimize negative impacts while maintaining employee proficiency and reducing organizational turnover.

However, significant gaps exist in the current research landscape. While much of the existing literature on shift work focuses predominantly on health deficits associated with shift workers, there is relatively limited research examining shift workers’ perceptions of rotational work systems from a psychological perspective. Furthermore, the majority of studies investigating shift workers’ perspectives employ quantitative methodologies (Aderemi et al., 2019; Kedzierska et al., 2019; Idowu Olanrewaju et al., 2019; Ihsan and Salami, 2020; Hirsh et al., 2022). To comprehensively explore shift workers’ experiences and perspectives, qualitative research approaches are essential for gaining deeper phenomenological understanding of the psychosocial mechanisms underlying their experiences.

This research gap is particularly pronounced in specific geographical and industrial contexts. In the Kingdom of Saudi Arabia, the number of shift workers has increased substantially over recent decades. Moreover, existing studies have predominantly focused on healthcare sectors rather than other critical industries such as petroleum and petrochemicals. Therefore, this research aims to explore the perceptions of shift workers toward working in rotational shifts through the lens of the JD-R model, with particular emphasis on workers in the petrochemical sector whose perspectives may differ significantly from those of healthcare workers. The significance of this research lies in its potential to contribute valuable, in-depth psychological knowledge specific to the Saudi petrochemical industry, which can inform future organizational development and policy decisions.

## 2 Literature review

Shift work, particularly rotational shift work, is essential in many businesses that require 24-h coverage, such as healthcare and emergency services, as well as manufacturing and hospitality. Because of the nature of such professions individuals are required to work beyond the usual nine-to-five schedule, which affects different aspects of their lives. Despite its widespread use, the effects of rotating shift work on individual health, safety, work-life balance, and occupational effectiveness are complex and poorly understood. To organize this body of evidence, the present review draws on the Job Demands-Resources (JD-R) model (Demerouti et al., 2001) as a guiding framework. The JD-R model distinguishes between job demands, which are aspects of work that require sustained physical or psychological effort, and job resources, which are aspects that support goal achievement, reduce demands, and stimulate personal growth (Demerouti, 2025). As will be shown, the four domains reviewed below map closely onto these two processes: health and safety consequences reflect the demand-strain pathway, while occupational and work-life balance outcomes reflect the interplay between demands and available resources.

Recent theoretical developments have continued to refine the JD-R model beyond its original formulation. Demerouti (2025) revisits the model alongside conservation of resources theory to explain contemporary patterns of employee well-being and job design, underscoring that the demands and resources most salient to workers are not fixed but are shaped by the broader occupational and institutional context in which work takes place. This contextual sensitivity is particularly relevant to the present study: much of the empirical JD-R literature has been generated in Western, healthcare-dominated settings, and the model’s core constructs have rarely been examined in relation to religious or culturally specific obligations. Extending the model to a non-Western, non-healthcare population, as undertaken here, therefore responds directly to calls for greater attention to how cultural context shapes the demands and resources experienced by workers.

### 2.1 Shift work and health

A growing body of literature has investigated the health issues associated with working in shifts. According to Knutsson (2003), sleep issues may play a role in shift workers’ health problems. The health effects of sleep deprivation are currently unclear; hence, some studies indicate that sleep deprivation may affect glucose tolerance (Spiegel et al., 1999). Previous research has established that rotational shifts with long hours can lead to increased employee turnover. Due to the work stress associated with fewer hours of sleep at night, as Wang and Yuan (2018) established, these factors can be major reasons for hospital care workers to think of quitting.

Another key finding is that rotational shift work can lead to insomnia (Khan et al., 2020). Furthermore, tiredness due to lack of sleep is linked to a higher likelihood of on-the-job and off-the-job accidents and a greater error ratio. Gold et al. (1992) demonstrated that rotational shift workers experience biological rhythms phase misalignment while being on duty, likely to result in attention failures. Recent longitudinal evidence reinforces these concerns, as shift workers sleep on average 72 min less than day workers, with sleep duration after night shifts falling well below recommended thresholds, and these deficits worsen significantly over time (Seibt et al., 2025). A parallel qualitative synthesis similarly found that shift work disrupts the alignment between the external environment and the body’s circadian rhythm, leading to chronic sleep difficulties and a broad decline in health (Benton et al., 2025). From a psychological standpoint, these health consequences are consistent with the JD-R model’s demand-strain pathway, whereby chronic exposure to high job demands, in this case disrupted sleep and irregular schedules, progressively depletes workers’ psychological and physical resources, increasing vulnerability to both physical illness and mental health difficulties.

### 2.2 Shift work and safety

To date, there has been little research into rotational shift work and its effect on workers (Knauth and Hornberger, 2003), and therefore there is an urgent need to identify shift systems that create the fewest health problems for workers, and to study rotational systems with the fewest consecutive night shifts, since night shifts can disrupt circadian physiological mechanisms (Harma, 2000).

One such finding in the literature is that shift workers’ circadian synchronization is unfavorably impacted due to inadequate deep sleep (Di Muzio et al., 2019). However, Haus and Smolensky (2006) suggest that to avoid circadian desynchronization, shift systems should contain no more than four consecutive night shifts, and include at least 1 day off in between for physiological recovery. In line with the work of Folkard and Tucker (2003), in order to reduce the overall danger of shift systems, we should evaluate the number of consecutive night shifts, their duration, and the provision for rest between them (Folkard and Tucker, 2003).

Researchers have attempted to assess the impact of shift work and sleep disturbance on commuting; for example, Rogers et al. (2001) evaluated shift workers and regular workers to identify any association between shift work and commuting risks and issues. The main variables studied were commuting distance, tiredness of workers, and commuting time, particularly on night shifts, and findings revealed that shift workers were more vulnerable to traffic accidents compared to regular workers. In addition, Boivin et al. (2022) demonstrated a higher incidence of severe fatigue at night, where results showed that the odds of severe tiredness were nine times higher in a group of truck drivers during the first night shift compared to day and evening shifts. Psychologically, these safety risks can be understood as a consequence of accumulated cognitive resource depletion, a process central to the JD-R model, whereby sustained exposure to demanding shift schedules erodes the attentional and executive functioning capacities needed to perform safely, both at work and during commuting.

### 2.3 Shift work and work-life balance

Prior studies have noted that night work is linked to spending more time with children, implying that night shifts allow parents to balance child care (Golla and Vernon, 2006). However, the downside of this is that shift employees tend to sleep less or experience more sleep difficulties than their daytime counterparts (Williams, 2001; Rosa and Colligan, 1997; Akerstedt, 2003). According to Shiffer et al. (2018), a rotating shift pattern is related to higher work-family and social conflict, possibly since it also negatively affects work-life balance. According to one study, a link exists between work-life balance and employee loyalty, satisfaction, and productivity (Dizaho, 2013). According to the findings, a good work-life balance has a positive effect on loyalty, productivity, and overall employee happiness. Sun’s research (Sun et al., 2018) shows that shift workers may be more likely to acquire abdominal obesity than other forms of obesity, and that permanent night shift employees can be 30% more at risk than rotating shift workers. These patterns persist in recent evidence, as a 2025 longitudinal study found that shift workers reported significantly greater work-life balance impairment than day workers, with effects compounding across years of shift work exposure (Seibt et al., 2025).

Within the JD-R framework, work-life conflict arising from rotational schedules can be understood as a demand that depletes personal and family resources, namely time, energy, and social connection, that are essential for psychological recovery and well-being outside of work. When these recovery resources are chronically unavailable, the buffering capacity of job resources such as schedule flexibility or social support becomes critically important.

### 2.4 Shift work and its occupational effects

According to Dall'Ora et al.’s (2016) findings, working overtime is linked to lower job performance, and rotating shift work is associated with poor job performance (Dall'Ora et al., 2016). Fitzpatrick’s findings also support previous studies showing that 12-h shifts are connected with less effective performance (Fitzpatrick et al., 1999). Ganesan underlined that alertness and performance were more affected at night compared to day shifts, with the highest harm reported at the conclusion of night shifts when assessments were delivered during an unfavorable circadian phase (Ganesan et al., 2019). Another study discovered that shift work leads to excessive daytime drowsiness, which can lead to sleep apnea. Sleep apnea is defined as the temporary cessation of breathing while sleeping. According to Silva et al. (2020), patients with sleep apnea often have poor cognitive and psychomotor performance. More recently, research among allied healthcare professionals in Saudi Arabia found that shift length and scheduling patterns were significant predictors of both burnout and reduced job satisfaction, with the majority of participants reporting at least moderate levels of burnout (Alotaibi et al., 2025).

This literature review provides an insight into the various implications of rotational shift work on health, safety, work-life balance, and occupational effectiveness, and reveals a complex tapestry of shift workers’ lived experiences, highlighting the multiple difficulties they face. Taken together, these four domains reflect the dual processes described by the JD-R model: rotational shift work generates substantial job demands that strain workers’ psychological and physical resources, while the presence or absence of meaningful job resources, including financial compensation, scheduling flexibility, social support, and career development opportunities, determines the extent to which workers can cope, recover, and remain engaged. While it is obvious that rotating shift work has a considerable influence on employees’ lives, this review highlights the need for more extensive and detailed investigation, and particularly for studies that prioritize the opinions and viewpoints of shift workers themselves, especially in non-Western industrial contexts where culturally specific demands such as religious obligations may constitute an additional and underexamined source of psychosocial burden.

## 3 Methodology

To achieve this study’s objectives and answer the main research question, a qualitative approach was undertaken. The nature of this study is exploratory research, which is appropriate when the available findings are limited or inadequate, and deeper investigation is necessary. The study was constructed as a qualitative investigation with several key characteristics: first, the research was conducted with shift employees at their workplace; second, data was gathered through interviews; and third, the research focused on acquiring and interpreting participants’ impressions and perceptions of rotational shift work.

### 3.1 Phenomenological orientation

This study is positioned within a hermeneutic, rather than descriptive, phenomenological tradition (van Manen, 1990). Whereas descriptive phenomenology seeks to bracket the researcher’s assumptions in pursuit of a pre-interpretive account of a phenomenon’s essential structure (Moustakas, 1994), hermeneutic phenomenology treats the researcher’s interpretive engagement with participants’ accounts as an inseparable and necessary part of understanding lived experience. This orientation was judged the better fit for the present study given the researcher’s insider positionality, described below, which shaped data collection and interpretation throughout rather than being held at a distance from it. Consistent with this orientation, the 12 core interview questions (Appendices 1, 2) were designed to elicit rich, first-person descriptions of participants’ concrete, everyday experience of rotational shift work, rather than abstract opinions or generalizations, in keeping with phenomenology’s central concern with the texture of lived experience itself. Braun and Clarke’s reflexive thematic analysis was selected as the analytic technique for organizing these accounts. Reflexive thematic analysis is not itself a discrete phenomenological method in the manner of Colaizzi’s or Giorgi’s structured procedures; however, Braun and Clarke (2019) explicitly position it as a flexible method rather than a methodology, one that can be, and frequently is, applied within a phenomenological theoretical framework, provided the researcher’s interpretive lens and assumptions are made explicit rather than treated as neutral. The present study adopts this pairing: phenomenology supplies the theoretical orientation toward lived experience and the researcher’s situated interpretation of it, while reflexive thematic analysis supplies the systematic procedure for moving from transcripts to themes. The reflexive journal and critical self-examination described in the following section functioned as this study’s bracketing practice, making the researcher’s prior assumptions visible and open to challenge throughout data collection and analysis, rather than assuming they could or should be eliminated entirely. Read together, the three themes reported below are intended to capture not only discrete categories of experience but a shared underlying structure, namely that rotational shift work in this cultural setting is lived as a continuous negotiation between externally imposed schedule demands and the personal, familial, and religious resources workers draw upon to remain whole across a fragmented working life.

### 3.2 Research context and positionality

The study was conducted within Saudi Arabia’s petrochemical sector for several significant reasons. Primarily, as a Saudi national, the researcher aims to contribute to the future development of business in the Kingdom of Saudi Arabia. Additionally, the researcher’s personal experience of working in a rotating shift environment provided insider knowledge of rotational working systems and insight into the challenges faced by shift employees in this context. It is important to acknowledge, however, that this insider positioning carries both advantages and limitations. While familiarity with the context enabled deeper rapport with participants and more nuanced interpretation of their accounts, it also required ongoing reflexive awareness to guard against the risk of confirming pre-existing assumptions rather than allowing new meanings to emerge from the data. Throughout the research process, the researcher maintained a reflective journal and engaged in regular critical self-examination to ensure that emerging themes were grounded in participants’ own words and experiences rather than in the researcher’s prior expectations. This reflexive stance is consistent with the principles of qualitative phenomenological inquiry, in which the researcher’s subjectivity is acknowledged and managed rather than eliminated (Braun and Clarke, 2019).

### 3.3 Sampling and participants

Given this contextual foundation, a purposive sampling technique was employed for participant selection. This technique was chosen to ensure that participating employees had a minimum of 3 years’ experience working rotational shifts, thereby excluding participants with limited rotational shift experience. The sample size of 16 participants was determined on the basis of data saturation, a widely accepted criterion in qualitative research whereby sampling continues until no new codes or themes emerge from the data (Braun and Clarke, 2019). This sample size is consistent with established guidance for phenomenological qualitative studies, in which smaller, purposively selected samples yield richer and more analytically meaningful data than larger, randomly selected ones (Creswell, 2014).

The 16 employees interviewed all worked in rotating shifts, and represented a variety of ages, work experience levels, and job titles. Participants were strategically divided into two groups: the first consisted of eight individuals in non-leadership roles, in order to gather broad information from frontline employees, while the second comprised eight participants in supervisory roles to obtain more detailed, management-perspective information. All 16 participants were male; no female employees were represented in the sample.

### 3.4 Data collection

For data collection, phenomenological interviews were selected as the primary method, as they are among the most well-known strategies for investigating perceptions. Semi-structured interviews were conducted to achieve the research objectives, as they allow for guided conversation while maintaining flexibility for follow-up questions. The interviews took place at participants’ workplaces in convenient, confidential, and preferred locations. Each semi-structured interview contained 12 core questions (provided in Appendices 1, 2), which introduced participants to the study topic and were followed by contextual questions based on participant responses and engagement. Following data collection, the researchers independently transcribed all recordings to provide deeper and more comprehensive understanding. Participant quotations presented throughout the findings are identified by a number in brackets, for example [12], which refers to the anonymized participant identifier assigned at the point of data collection.

### 3.5 Ethical considerations

This study was conducted in accordance with the ethical principles governing research with human participants. Ethical approval was obtained from the Research Ethics Committee (REC) of the Faculty of Economics and Administration (FEA), King Abdulaziz University (Approval No. REC/2025/0029; approved on 21 November 2025). Throughout this process, comprehensive ethical protocols were implemented to ensure participant comfort and data security. Participants were informed about the confidential nature of interview materials and advised that conversations would be recorded for scientific purposes only. Written informed consent was obtained from all participants prior to their involvement in the study. All data, including interview recordings and notes, were password-secured and stored on a secure computer accessible only to the researcher. This anonymity encouraged participants to express their thoughts more openly, resulting in the acquisition of rich, detailed data.

### 3.6 Data analysis and trustworthiness

For data analysis, the study employed a six-step thematic analysis approach as outlined by Braun and Clarke (2019), which included familiarization with the data, code generation, theme construction, examining prospective themes, defining and naming themes, and report production. In practice, the researcher first read and reread transcripts multiple times to become thoroughly acquainted with the data. Subsequently, codes were assigned to text fragments that indicated specific thoughts or concepts, with this process repeated until no new codes or ideas could be discovered. Following the coding phase, categories were identified through linkages between distinct codes, and subthemes and themes were created to group similar categories. The researcher then compared emergent categories against the original data to ensure accurate representation, after which subthemes and themes were assigned appropriate names, and a comprehensive analytical report was produced.

To ensure the trustworthiness of the findings, the study applied Lincoln and Guba’s (1985) four criteria of rigor for qualitative research. Credibility was addressed through prolonged engagement with the data, whereby transcripts were read and reread across multiple analytical sessions, and through the use of direct participant quotations throughout the findings to anchor interpretations in the raw data. Transferability was supported by providing rich, detailed descriptions of the research context, participant characteristics, and analytical process, enabling readers to assess the applicability of findings to comparable settings. Dependability was ensured through the maintenance of a clear and documented audit trail of analytical decisions, from initial coding through to final theme construction. Confirmability was addressed through the researcher’s reflexive practice, as described above, which aimed to make explicit the influence of the researcher’s positionality on the interpretive process and to ensure that findings reflected participants’ perspectives rather than researcher-imposed meanings.

### 3.7 AI assistance disclosure

During the preparation of this manuscript, AI-assisted tools were used to support the editing, language refinement, and structural organization of the text. All scientific content, including the study design, data collection, participant interviews, thematic analysis, interpretation of findings, and conclusions drawn, are entirely the work of the authors. The use of AI tools was limited to improving the clarity and academic presentation of ideas that originated solely with the authors. This disclosure is made in accordance with Frontiers in Psychology’s editorial transparency guidelines.

## 4 Results

The findings of this study give insight into the numerous effects of rotational shift work on employees’ health, work-life balance, and professional lives. Exploration of these aspects of shift employment reveals a complex picture complete with advantages and disadvantages. The study identified three primary themes that offer a comprehensive understanding of the impact of rotational shift work as presented in the conceptual framework in Figure 1. Interpreted through the Job Demands-Resources (JD-R) model, the findings reveal a consistent pattern whereby the demands generated by rotational shift schedules, including sleep disruption, social isolation, and career constraints, deplete workers’ psychological and physical resources, while certain job resources, notably financial incentives, scheduling flexibility, and collegial bonds, serve to buffer or partially offset these effects. First, the interpreted data revealed a significant influence on health, with participants regularly describing poor sleep, exhaustion, and trouble sustaining a healthy lifestyle. Second, work-life balance emerged as a matter of habit, emphasizing how individuals dealt with the complications of rotating shift employment. Finally, the study looked into the occupational impacts of rotating shift employment.

**Figure 1:**
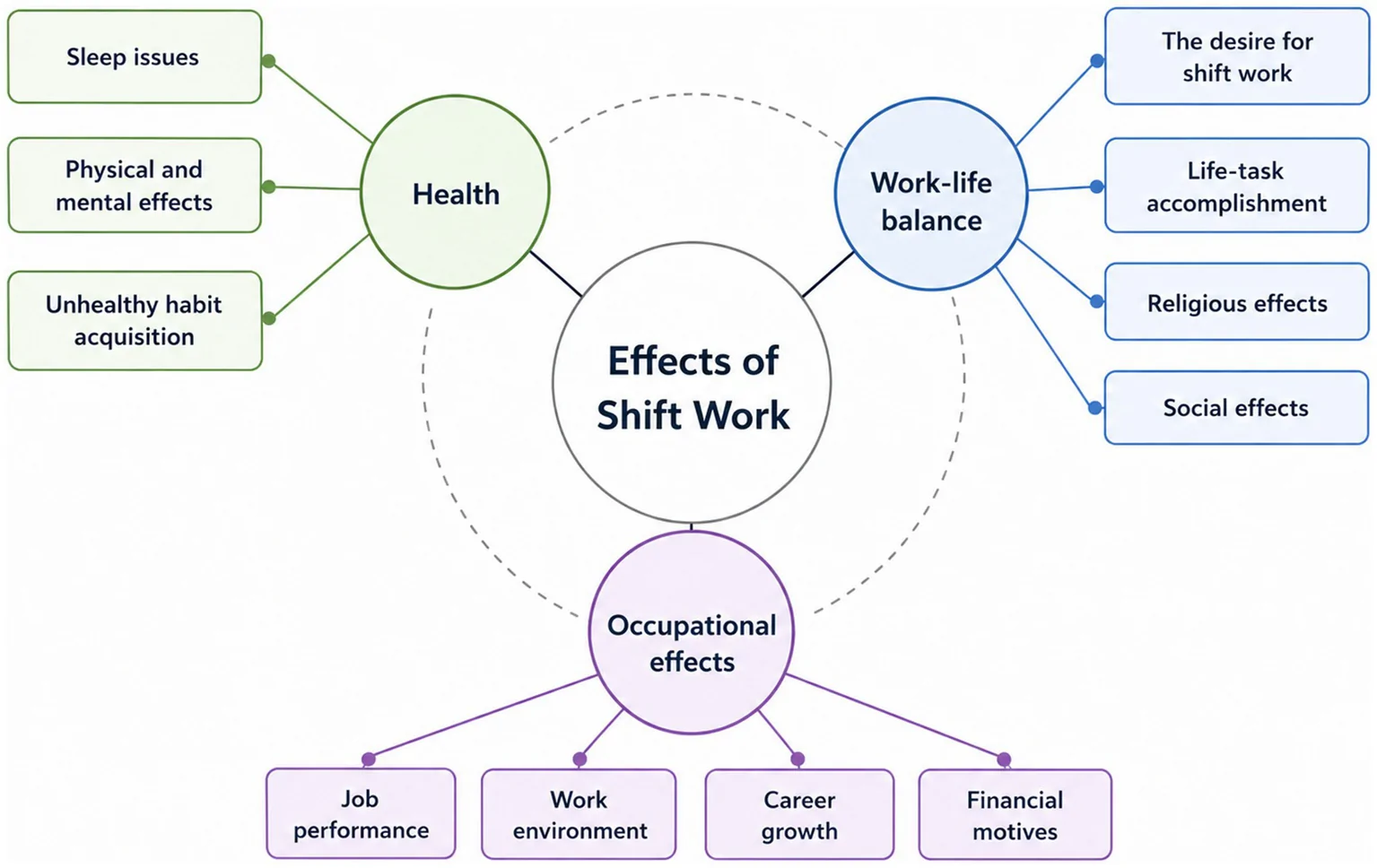
Themes and sub-themes identified from participants’ experiences of rotational shift work.

### 4.1 Health

A health theme was generated from the data, and analysis showed that sleep difficulties, physical and mental deficits, and unhealthy habits were the three most frequent subthemes associated with the negative health effects of shift work. These findings reflect a cumulative depletion of workers’ physical and psychological resources under chronic scheduling demand, a pattern examined in more depth in the Discussion below.

#### 4.1.1 Sleep issues

Due to the importance of health in the lives of shift workers, health issues linked with working in rotating shifts had a monopolizing influence on all other components. Participants’ comments consistently pointed to sleep as a crucial element that influenced them on several levels.

*“Number one of the health issues I have faced is the lack of regular sleep and associated problems, such as insomnia and circadian rhythm disruption, which both lead to physical health problems and reduced physical activity. [12]”*

As indicated by one of the participants, illness induced by sleep patterns switching from morning to night and vice versa could lead to migraine and other related conditions. Rains et al. found that certain types of headache, including hypnic, cluster, and migraine headaches, have chronobiologic patterns that may be driven by the disturbance of regular sleep in those who are predisposed to headaches, for example by sleep deprivation or schedule changes (Rains et al., 2008).

*“Sleep disruptions and the continuous reversal of sleep times have a continual effect on the diurnal body rhythms, and one wakes up feeling sluggish and not knowing whether it is night or day. The difficulty lies in changing from night sleeping to daytime sleeping and back again, so sleep rhythms are perpetually disturbed. Sleep pattern disturbance is one of the strongest factors in migraine conditions, which have no treatment to this day. [14]”*

The research revealed considerable health concerns among shift workers, with sleep troubles being highlighted as major issues. The inconsistent sleep patterns caused by rotational shifts, in particular, had a significant influence on individuals’ physical health and total activity levels. Furthermore, the constant shift in sleep periods disturbed their biological clocks, increasing their risk of migraines and other health problems.

#### 4.1.2 Physical and mental effects

Reactions to shift work included exhaustion and a lack of physical energy for performing regular human tasks, which may also impact health in general because of the inability to actively engage in life. One response expressed going to work as an exhausting journey that has to be done every day.

“*I am physically exhausted by the nature of my work, and I work a long way from the city I live in. I drive 140 km to get to work each day, and do the same journey back after my shift. It is really exhausting. I never seem to get enough rest or sleep, and it is taking a toll on my body. I feel permanently exhausted and in pain. [5]”*

Daily long-distance commuting and shift work were found to cause physical tiredness and a lowered ability to perform routine tasks, thereby compromising participants’ overall health. Inadequate rest increases physical discomfort and exhaustion, pointing to negative physical and mental health effects.

#### 4.1.3 Unhealthy habit acquisition

Some participants expressed dissatisfaction with their inability to engage in regular healthy activities like working out at a gym or participating in physical sports, and they undoubtedly blamed shift work for their inability to set up consistent times for these activities.

*"I have these goals to stick to a nutritious diet and go to the gym consistently, but my work shifts make it difficult for me to sort out regular times to achieve these goals, and I am hardly ever able to get to the gym and work out, which is really frustrating me. [4]"*

Additionally, one participant was unhappy with his diet as a result of working late at night, which made him more likely to consume fast food with low nutritional value owing to a lack of easy access to regular home-cooked meals.

*"Uncontrollable weight gain and trouble visiting the gym, particularly after work hours. Feelings of hunger and the need to eat get so much worse during night shift hours, which really bothers me, as I end up eating junk food because there are not many options at these times. [16]"*

Shift workers’ schedules are inconsistent and often unpredictable, making it difficult to maintain consistent practices of exercise and healthy eating. This might result in weight gain, difficulty managing appetite, difficulty making good dietary choices, and a sense of discontent about general health. These results correspond to previous research, which indicates that shift work might affect dietary patterns, contribute to poor eating habits, and have a negative impact on physical activity levels (Kecklund and Axelsson, 2016). Taken together, the three health subthemes reflect a compounding cycle of demand-driven resource depletion in which disrupted sleep undermines physical health, physical exhaustion limits engagement in restorative activities, and the absence of healthy routines further erodes the personal resources workers need to cope with the demands of their roles.

### 4.2 Work-life balance

The desire to work in shifts can be linked to a variety of factors, including personal situations as well as financial incentives. This theme captures both the demands that rotational schedules impose on participants’ personal lives and the resources, such as financial reward and scheduling flexibility, that some drew upon to adapt; the interplay between these is developed further in the Discussion.

#### 4.2.1 The desire for shift work

According to the responses received from the participants, there are two distinct perspectives on why shift workers choose to work in rotational shifts. Some shift workers stated that they work in rotational shifts due to a lack of other job opportunities or qualifications for traditional careers.

*"I work in rotational shifts due to a lack of alternative possibilities due to my qualifications. If I could turn back the clock, I would try hard to get a regular career that did not involve shift work, even though it is higher paid in order to compensate for the disadvantages. [9]"*

*"I think employees should continually look for more opportunities to work typical daytime hours and view shift work as a temporary phase in their lives. [13]"*

#### 4.2.2 Life-task accomplishment

There are a few advantages to working in rotational shifts when it comes to completing life tasks, such as personal flexibility. Some participants mentioned that working in rotational shifts allowed them to avoid the crowded hustle and bustle of rush hour and evenings, when most people are out and about after finishing work.

*"One of the best things about working rotational shifts is that you can do things during the day and avoid the crowds because most people are at work. This is sometimes useful when you want to travel in relative peace, which you can do during daytime office hours, while everyone else is at work. [6]"*

*"Working in rotating shifts has its benefits, one of which is that I can complete all of my responsibilities and work for government agencies in the morning on my rest days in the middle of the week. Being able to deviate from my typical schedule and do important things in the middle of the week inspires me. [7]"*

#### 4.2.3 Religious effects

For the participants interviewed, rotating shift work resulted in consequences for both their religious life and their work-life balance. For example, the variable nature of shift work could make it challenging to fulfil religious obligations, such as keeping to specific prayer times.

*"There are various religious consequences, such as having to postpone prayers and not being able to pray at specific times with other people in the mosque. Ramadan is severely disrupted, preventing you from conducting supererogatory prayers, such as the Tarawih prayer. [12]"*

*"I find it difficult to pray on Fridays (Juma'a), which has a negative impact on my spirituality. I also think that being unable to visit with my family is an expression of religious inadequacy. [4]"*

These responses highlight the religious effects and challenges faced by shift workers in maintaining their spiritual practices and participating in religious events. The disruption of prayer routines, limitations on attending mosque services, and difficulties in observing religious traditions with family can impact their sense of religious fulfillment and contribute to work-life balance concerns from a religious perspective.

#### 4.2.4 Social effects

Based on the responses gathered from participants, the social effects of working rotational shifts are significant factors impacting work-life balance. Participants highlighted that one of the most negative aspects of working in rotational shifts is the sense of social isolation and the inability to attend social events.

*"The most negative aspect of working rotating shifts is the social isolation and inability to attend social events. I feel disappointed when I have trouble attending social events due to the conflict with working hours. [7]"*

*"I hate being away from the family, and sometimes they do not know when your free time and rest days are, which makes it difficult to gather with them. Socially, I do not like not being able to attend family and friends' gatherings if my shifts are on Fridays and Saturdays. [16]"*

These responses highlight the social challenges faced by shift workers in terms of social isolation, missed social events, difficulties in coordinating family time, and the inability to attend weekend gatherings. The religious and social subthemes together reveal a dimension of psychosocial demand that is particularly salient in the Saudi Arabian context, where religious observance and family cohesion are central to cultural identity.

### 4.3 Occupational effects

The theme of occupational effects associated with rotational shift work encompasses various work-related consequences. These subthemes illustrate how shift work simultaneously constrains career development while offering resources, such as experiential learning, that sustain motivation, a tension explored further below.

#### 4.3.1 Job performance

It appears that some participants perceived rotational shift work as an opportunity to gain valuable professional experience. Participants mentioned that working in rotational shifts exposed them to a variety of situations and challenges that might contribute to their professional growth.

*"Due to the absence of limitations and the increased likelihood of issues and occurrences that will require experience to handle in the future, rotational shift workers build a lot more experience. [11]"*

*"In my opinion the shift worker obtains more professional experience because he will be the one to make the decision and come up with the solution when a problem arises during the night shift. [7]"*

#### 4.3.2 Work environment

Responses that were relevant to the work environment subtheme revealed conflicting opinions regarding the work environment of rotational shift work. Some participants expressed a preference for regular work environments over shift work environments.

*"Regular work environments are preferable to shift work environments because regular work is consistently clearer in terms of communication and misunderstandings between shift employees. Decisions are frequently made by regular workers in the morning, making regular work environments better in terms of communication. [6]"*

*"Since an individual's emotional state is at its most regulated and calm during daytime hours, the work environment for regular employees appears to be better than for rotational shift workers. [7]"*

*"The team spirit is greater in a rotating work environment since everyone is with one another for extended periods of time, which improves their understanding of one another and fosters collaboration. [16]"*

#### 4.3.3 Career growth

Participants expressed some of their concerns about the limitations and challenges to career growth that are inherent in shift work.

*"The disadvantage is that you work hard and suggest improvements, but no one pays you any attention, because the shift employee is only a worker from management's perspective. [7]"*

*"It is difficult to achieve promotion when you are doing shift work. We find it much harder to reach higher positions compared to normal regular daytime workers. The regular workers are much better off in terms of gaining experience and the evidence is that most of the successful and senior staff are regular daytime employees. Also, shift work affects the mind, health, and body, and employees on rotating shifts cannot easily rise up the ranks. [12]"*

#### 4.3.4 Financial motives

There were some intriguing viewpoints that contradicted the widespread belief that working in rotational shifts results in greater financial rewards.

*"My first reason for choosing to work rotating shifts was the very attractive salary, as it is much higher paid than day work; there is nothing attractive about shift work except the financial aspect. [5]"*

*"Employees working in rotating shifts often accept job offers in a rush without thinking of the bigger picture. Maybe they are young job seekers trying to find a well-paid job, but they do not realize that their salaries will not increase at anything like the same rate as those of regular daytime workers. [5]"*

Across all four occupational subthemes, the JD-R model helps to explain why shift workers experience such mixed outcomes: where job resources such as financial rewards and experiential autonomy are present, they sustain engagement and provide motivation, but where demands such as limited visibility, career barriers, and relationship constraints dominate, they erode the professional resources workers need to grow and advance within their organizations.

## 5 Discussion

It is worth noting that our participants’ replies did not correspond to the concerns about safety that tend to be addressed in the current literature. This disparity could be related to the fact that safety issues are mainly an organizational responsibility rather than a direct concern of the shift employees themselves. To prevent fatigue-related incidents, organizations are expected to guarantee proper rest periods, implement safety trainings, and limit work hours (Steffey et al., 2023). From a JD-R perspective, this finding is theoretically meaningful: safety is best understood as an organizational-level resource, one that is designed, maintained, and monitored by management rather than experienced as a personal demand or resource by individual workers. A plausible explanation for why safety did not surface as a personal concern is that petrochemical operations in Saudi Arabia are typically governed by highly formalized, mandated occupational health and safety systems, so that risk mitigation is experienced by workers as an institutional given rather than something requiring individual vigilance or discretionary effort. Under this interpretation, safety functions as a background organizational resource that is largely invisible to workers precisely because it is functioning as intended, and it may only become salient as a personal concern when such systems fail or are perceived as inadequate. Future research could test this interpretation directly by asking shift workers to describe who they consider responsible for workplace safety.

### 5.1 Health

The findings presented above show that shift work has a negative impact on health, notably in terms of sleep, physical well-being, and the maintenance of healthy habits. These findings are consistent with previous research, which has widely documented the difficulties shift workers have in controlling their sleep and overall health (Knutsson, 2003). Sleep difficulties, such as insomnia and misaligned biological clocks, for example, have been related to shift work and can contribute to other health issues, such as migraines (Rains et al., 2008). Shift work can have a negative impact on both physical and emotional health, according to the study findings. Due to their work schedules, participants expressed fatigue, a lack of energy, and difficulty engaging in normal activities. This is consistent with earlier research, which has shown exhaustion and sleep deprivation as common difficulties among shift workers, both of which can contribute to physical and mental health issues (Akerstedt, 2003; van Drongelen et al., 2011). The research additionally found that shift work can make it difficult to maintain healthy behaviors like exercising regularly and eating a balanced diet. This is consistent with previous research indicating that shift employment has a negative impact on diet and physical activity (Kecklund and Axelsson, 2016; Antunes et al., 2010).

Interpreted through the JD-R model, these health findings reflect the operation of the demand-strain pathway in particularly acute form. The irregular and rotating nature of shift schedules constitutes a sustained, chronic job demand that continuously depletes workers’ physical and psychological energy reserves. Unlike fixed-schedule workers who can establish stable sleep, nutrition, and exercise routines that replenish these reserves, rotational shift workers face a structural barrier to recovery: their schedules prevent the very restorative behaviors that would allow them to rebuild depleted resources. This creates a self-reinforcing cycle of depletion in which poor sleep leads to fatigue, fatigue undermines the motivation and capacity to exercise or eat well, and the resulting decline in physical health further reduces workers’ resilience to the demands of their roles.

### 5.2 Work-life balance

People chose to work in rotational shifts for a variety of reasons, including limited career opportunities and financial incentives, according to the findings. Prior research has also shown that economic necessity, job security, and higher pay can all impact an individual’s decision to work in shifts (Barton, 1994; Nabe-Nielsen et al., 2011). Furthermore, the perspective of shift work as a brief period in one’s life shows the adaptive techniques that some people may use to deal with the obstacles that such work arrangements present. In addition to the challenges, some participants recognized the benefits of working in rotational shifts, notably in terms of personal flexibility and responsibility fulfillment. Previous research has identified that rotational shift work allows employees to travel in less crowded conditions and engage in daytime activities while working night shifts or on their mid-week days off (Folkard et al., 2005).

A particularly significant and novel finding of this study, however, concerns the religious and social dimensions of work-life balance among Saudi shift workers, which have received little attention in the existing literature. Participants consistently described the disruption of prayer schedules, the inability to attend Friday congregational prayers, the interruption of Ramadan observance, and the difficulty of fulfilling family visitation obligations as sources of profound psychosocial distress. Within the JD-R framework, these culturally specific obligations represent a distinct category of personal resource, namely spiritual fulfillment, religious identity, and family connectedness, whose depletion under rotational shift conditions generates a form of demand-induced resource loss that is qualitatively different from the sleep and fatigue burdens documented in Western literature. This finding makes an important contribution to the JD-R literature by demonstrating that the model’s resource construct must be understood as culturally situated.

### 5.3 Occupational effects

The study’s findings offer important insights into the occupational implications of rotational shift work on factors such as job performance, work environment, career advancement, and financial considerations. While some participants reported excellent professional development and decision-making opportunities, others voiced complaints about the work environment, the limited opportunities for career progression, and the potential negative influence on professional relationships (Costa, 2003; Demerouti et al., 2004). In terms of job performance, findings suggest that shift work can improve decision-making and problem-solving skills by exposing workers to a variety of scenarios and problems (Wong et al., 2010).

From a JD-R perspective, the occupational findings are particularly revealing because they demonstrate the model’s motivational pathway operating alongside the demand-strain pathway. Participants who reported enhanced decision-making autonomy and experiential learning during night shifts were effectively describing the activation of job resources, specifically autonomy, skill variety, and professional challenge, that sustained their engagement and motivation despite the demanding nature of their schedules. Conversely, participants who reported career invisibility, limited management contact, and restricted promotion prospects were describing the erosion of career-related resources, including recognition, feedback, and developmental opportunity. This asymmetry suggests that the occupational consequences of shift work are not uniformly negative, but are instead shaped by the degree to which workers can access job resources that buffer the career-limiting demands of their scheduling arrangements.

## 6 Conclusion

This study contributes to the literature on rotating shift work by offering a comprehensive analysis of its impact on employee health, work-life balance, and occupational outcomes, interpreted through the theoretical lens of the Job Demands-Resources (JD-R) model. By applying the JD-R framework to the lived experiences of shift workers in Saudi Arabia’s petrochemical sector, the study advances understanding of how rotational scheduling generates chronic job demands that deplete workers’ psychological and physical resources, while simultaneously revealing how certain job resources, including financial incentives, scheduling flexibility, and collegial bonds forged during extended night shifts, serve to partially buffer these effects.

A particularly significant theoretical contribution of this study lies in its identification of culturally specific demands that fall outside the scope of existing Western-oriented shift work literature. The disruption of Islamic religious obligations, including daily prayer schedules, Friday congregational prayers, and Ramadan observance, alongside the erosion of family visitation practices that carry deep cultural and spiritual significance in the Saudi context, represents a distinct category of psychosocial resource loss that the JD-R model has not previously been applied to examine. These findings extend JD-R theory by demonstrating that the resource construct must be understood as culturally situated, and that models of occupational well-being developed primarily in Western healthcare contexts require meaningful adaptation when applied to non-Western, non-healthcare populations. Beyond this cultural contribution, the study’s contrast between frontline and supervisory perspectives, drawn from within a single organizational context, offers a further point of departure from the wider shift-work literature, which has rarely compared how the same rotational demands and resources are experienced across hierarchical position; several findings reported above, notably around career visibility and professional recognition, took on a different character depending on whether the participant occupied a frontline or supervisory role.

The results highlight practical implications for organizations and policymakers. Employers are encouraged to adopt proactive strategies that foster healthy work environments, including initiatives to support sleep hygiene, mental well-being, and physical health. Beyond these general measures, organizations operating rotational shift systems in Muslim-majority contexts should give explicit consideration to the scheduling of religious obligations, including the accommodation of Friday prayers, the adjustment of shift patterns during Ramadan, and the provision of designated prayer facilities and times within the workplace.

Nevertheless, several limitations must be acknowledged. The reliance on self-reported data may introduce bias, while the relatively small qualitative sample of 16 participants limits the generalizability of findings. Furthermore, the study did not account for individual traits, coping mechanisms, or differences across job types, which could influence experiences of shift work. It should also be noted that the study was conducted by a researcher with personal experience of rotational shift work in the Saudi petrochemical sector, and while reflexive practices were employed throughout to manage the influence of this positionality, the possibility of interpretive bias cannot be entirely excluded. A further limitation is that all 16 participants were male, which restricts the transferability of the findings to female shift workers and means the study cannot speak to gender-specific demands and resources within this workforce.

Future research should address these limitations by employing larger and more diverse samples, exploring varied industries, and incorporating longitudinal and experimental designs to capture causal relationships. Future studies should also examine the experiences of female shift workers in Saudi Arabia and other Gulf Cooperation Council countries, where gender-specific cultural expectations may generate a distinct set of demands and resources not captured in the present study. Cross-cultural comparative studies examining shift workers across Muslim-majority and non-Muslim-majority contexts would further advance understanding of how cultural and religious factors mediate the relationship between rotational scheduling and psychosocial well-being.

## Data Availability

The original contributions presented in the study are included in the article/Supplementary material, further inquiries can be directed to the corresponding author.
